# T_2_ mapping of the meniscus is a biomarker for early osteoarthritis

**DOI:** 10.1007/s00330-019-06091-1

**Published:** 2019-03-19

**Authors:** Susanne M. Eijgenraam, Frans A. T. Bovendeert, Joost Verschueren, Jasper van Tiel, Yvonne M. Bastiaansen-Jenniskens, Marinus A. Wesdorp, Kazem Nasserinejad, Duncan E. Meuffels, Jamal Guenoun, Stefan Klein, Max Reijman, Edwin H. G. Oei

**Affiliations:** 1 Department of Radiology & Nuclear Medicine, Erasmus MC, University Medical Center, Dr. Molewaterplein 40, room Nd-547, 3015 GD Rotterdam, The Netherlands; 2 Department of Orthopedic Surgery, Erasmus MC, University Medical Center, Rotterdam, The Netherlands; 3 Department of Orthopedic Surgery, Rijnstate Hospital, Arnhem, The Netherlands; 4 Department of Hematology, Erasmus MC, University Medical Center, Rotterdam, The Netherlands; 5 Department of Radiology, Cambridge University Hospitals, Cambridge, UK; 6 Department of Medical Informatics, Erasmus MC, University Medical Center, Rotterdam, The Netherlands

**Keywords:** Knee, Meniscus, Osteoarthritis, Magnetic resonance imaging

## Abstract

**Purpose:**

To evaluate in vivo T_2_ mapping as quantitative, imaging-based biomarker for meniscal degeneration in humans, by studying the correlation between T_2_ relaxation time and degree of histological degeneration as reference standard.

**Methods:**

In this prospective validation study, 13 menisci from seven patients with radiographic knee osteoarthritis (median age 67 years, three males) were included. Menisci were obtained during total knee replacement surgery. All patients underwent pre-operative magnetic resonance imaging using a 3-T MR scanner which included a T_2_ mapping pulse sequence with multiple echoes. Histological analysis of the collected menisci was performed using the Pauli score, involving surface integrity, cellularity, matrix organization, and staining intensity. Mean T_2_ relaxation times were calculated in meniscal regions of interest corresponding with the areas scored histologically, using a multi-slice multi-echo postprocessing algorithm. Correlation between T_2_ mapping and histology was assessed using a generalized least squares model fit by maximum likelihood.

**Results:**

The mean T_2_ relaxation time was 22.4 ± 2.7 ms (range 18.5–27). The median histological score was 10, IQR 7–11 (range 4–13). A strong correlation between T_2_ relaxation time and histological score was found (*r*_s_ = 0.84, CI 95% 0.64–0.93).

**Conclusion:**

In vivo T_2_ mapping of the human meniscus correlates strongly with histological degeneration, suggesting that T_2_ mapping enables the detection and quantification of early compositional changes of the meniscus in knee OA.

**Key Points:**

*• Prospective histology-based study showed that in vivo T*_*2*_ *mapping of the human meniscus correlates strongly with histological degeneration.*

*• Meniscal T*_*2*_ *mapping allows detection and quantifying of compositional changes, without need for contrast or special MRI hardware.*

*• Meniscal T*_*2*_ *mapping provides a biomarker for early OA, potentially allowing early treatment strategies and prevention of OA progression.*

**Electronic supplementary material:**

The online version of this article (10.1007/s00330-019-06091-1) contains supplementary material, which is available to authorized users.

## Introduction

The pivotal role of the meniscus in knee osteoarthritis (OA) has attracted considerable attention among researchers for decades. Not only is meniscal damage a radiological sign of OA—up to 91% of the patients with symptomatic knee OA have coexisting meniscal tears [1]—a torn meniscus is also one of the strongest risk factors for the development and progression of knee OA [2–5]. Although the complex role of meniscal tissue composition in the etiology of meniscal tears and the subsequent development of knee OA is not entirely clear, it has become increasingly evident that the menisci play a critical role in the long-term health of the knee joint.

Hence, the ability to objectively assess meniscal tissue quality and composition is of key importance, particularly in patients at risk for developing knee OA [2]. In order to study the etiology of meniscal tears and meniscal degeneration in knee OA development and progression and to allow early interventions and prevention of progression, changes in meniscal tissue composition need to be detected before gross morphological changes occur.

Using conventional magnetic resonance (MR) imaging, measuring such changes in meniscal tissue composition prior to surface breakdown is challenging. Recent developments in quantitative MR imaging techniques, such as T_2_ mapping and T_1_rho, have made great progress in addressing this challenge [6, 7]. Among quantitative MR imaging techniques, T_2_ mapping is the most commonly used in knee OA research [8, 9]. Based on properties of biochemical tissue components, quantitative analysis of T_2_ relaxation times can reveal the composition of extracellular matrix, without the need for contrast or special MR hardware [6, 10]. Increased T_2_ relaxation times indicate damage to the collagen network and a decrease in water content, both signals of tissue degeneration [11].

Recent studies have shown the potential of T_2_ relaxation time as biomarker to quantify meniscal degeneration in patients with knee OA [6, 12–14], yet validation studies of meniscal T_2_ mapping are limited. Validation of T_2_ mapping using histological analysis (the gold standard for tissue changes) was performed in one previous study [7]. In that study, T_2_ mapping was performed ex vivo; however, it is unknown how well T_2_ measurements, obtained ex vivo, reflect the actual in vivo situation. To our knowledge, validation of in vivo meniscal T_2_ mapping, using histological analysis as reference test, has not been performed.

We aimed to validate in vivo meniscal T_2_ mapping in patients with knee OA by evaluating the correlation between T_2_ mapping and histological reference standards for meniscal degeneration.

## Materials and methods

### Study design and participants

Our prospective observational study was conducted between April 2016 and July 2017. Meniscal specimens were obtained from patients with primary end-stage knee OA undergoing elective total knee replacement surgery at our institution. Participants were selected consecutively. Study approval was granted by the institutional Medical Ethical Committee (MEC-2012-218), and written informed consent was obtained from all participants.

### Assessment of radiological knee OA

The assessment of radiological knee OA is described in Appendix 1 in the Supplementary Material.

### MR image acquisition

MR imaging was performed on a 3-Tesla (T) MR unit (Discovery MR750, GE Healthcare), 1 day prior to surgery. The MR imaging protocol included routine morphological knee sequences (proton density–weighted sequences in sagittal, coronal, and axial plane; T_2_-weighted sequences with fat saturation (Fat-Sat) in sagittal, coronal, and axial plane) and a sagittal 3D Fat-Sat fast spin-echo (FSE) T_2_ mapping sequence with multiple echoes. An 8-channel S&R rigid dedicated knee coil (GE Healthcare) was used. Sequence parameters are displayed in Table 1.

**Table 1:** MR imaging sequence parameters

MR imaging sequence parameters	
Scanner type	Discovery MR750, GE Healthcare
Scanner field strength	3 T
Sequence type	3D fast spin-echo fat suppression
Matrix (RO × PE)	288 × 192
Interpolated resolution (mm^2^)	0.5 × 0.8
Slice thickness/spacing	3/0
Number of slices	36
Number of echoes	5
TE (ms)	3.1; 13.4; 27.0; 40.7; 68.1
TE used for map reconstruction (ms)	3.1; 13.4; 27.0
FOV (cm)	15
Coil	8-channel S&R rigid knee coil, GE Healthcare
Scan time (mm:ss)	09:41

*T* tesla, *RO* readout, *PE* phase encoding, *TE* echo time, *FOV* field of view, *S&R* send and receive

### Harvesting of meniscal tissue and histological analysis

**Fig. 1:**
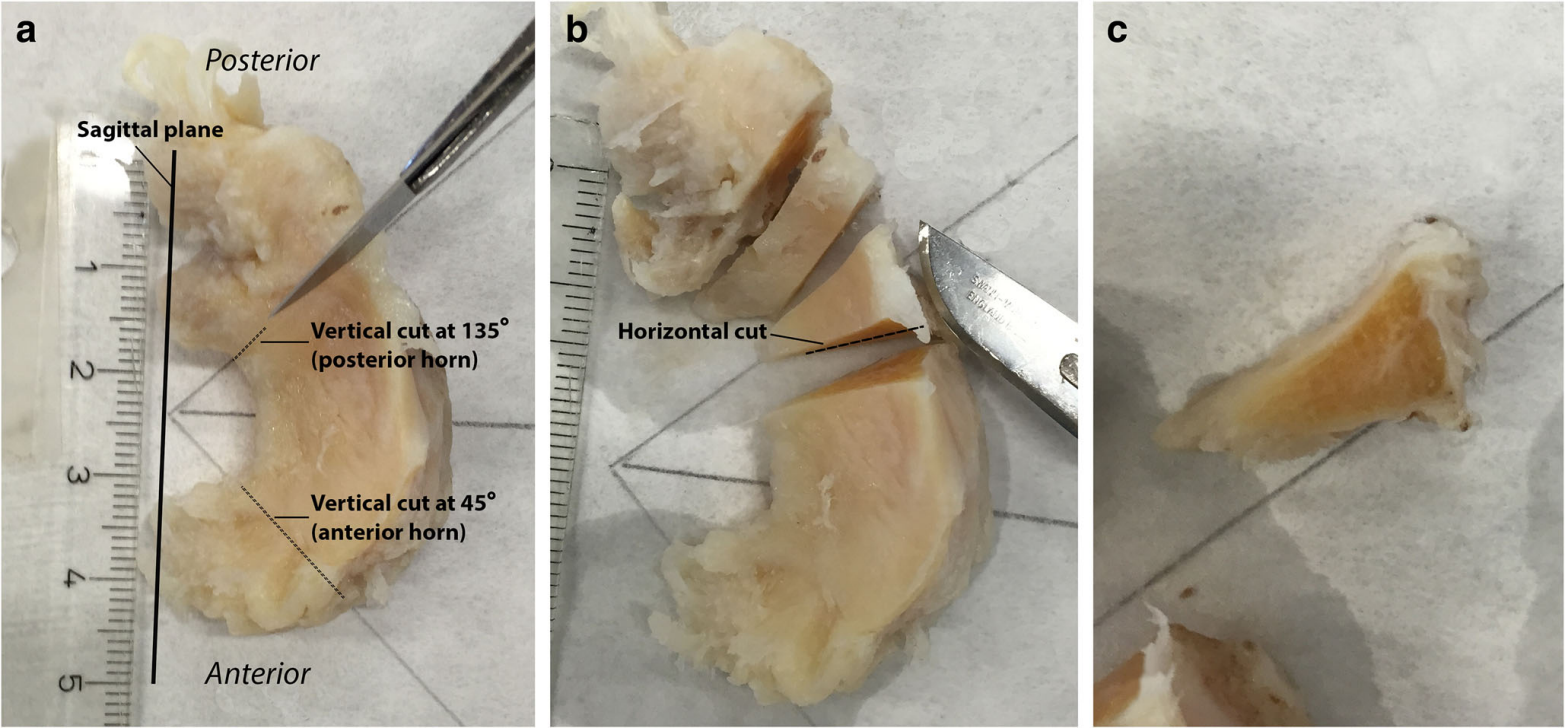
Preparation of meniscal samples. Example of a grossly intact lateral meniscus harvested during total knee arthroplasty in the left knee of a 59-year-old female with medial compartment knee OA (Kellgren and Lawrence grade 4). **a** Cutting the meniscus according to the method of Pauli et al: vertical cut. **b** Horizontal cut, from the inner rim to the vascular zone at a 30° angle relative to the tibial plateau. **c** Detail view of the vertical cut of the posterior horn

Meniscal specimens were obtained intraoperatively, during total knee replacement surgery. If present, both medial and lateral menisci were harvested; meniscal samples were stored in formaldehyde. Within 3 days of harvesting, menisci were cut in a standardized way according to Pauli et al [15] (Fig. 1). For each meniscus, the anterior horn and the posterior horn were processed. The menisci were cut at 45° (for the anterior horn) and 135° (for the posterior horn) angles relative to the sagittal plane (Fig. 1a). Meniscal samples were cut along two different planes: the vertical plane and the horizontal plane. The vertical section provided an overview of the longitudinally oriented collagen bundles and the tibial and femoral surfaces of the meniscus (Fig. 1c). The horizontal section, cut from the inner rim to the vascular zone at a 30° angle relative to the tibial plateau, revealed the parallel organization of the collagen bundles and matrix morphology (Fig. 1b).

The samples were fixed, dehydrated in alcohol, and infiltrated with paraffin. Next, meniscal samples were paraffin-embedded and sectioned using a microtome (MR2235, Leica-Biosystems) into 6-μm sections. To provide an overview of the overall tissue organization and to assess border integrity, cellularity, and cell morphology, sections were stained using hematoxylin and eosin. Safranin O-fast green and Alcian blue stains were used to evaluate proteoglycan content and mucoid degeneration, respectively. To assess collagen fiber organization, Picrosirius red stain was used. Stained sections were visualized using (polarized-) light microscopy (Olympus-BX50, Olympus-Optical) [16]. To assess the histological degree of degeneration, the validated, semi-quantitative Pauli score [15] was performed by two investigators with 4 years of experience in musculoskeletal research (Table 2). Both investigators were blinded to patient information and imaging outcomes. They examined all meniscal samples individually; in case of discrepancies, sections were assessed in consensus.

**Table 2:** Histological scoring system to assess meniscal degeneration by Pauli et al. The range of possible total scores is 0–18. This total score can be converted to a grade as follows: grade 1 = 0–4, grade 2 = 5–9, grade 3 = 10–14, grade 4 = 15–18. Grade 1 represents normal tissue, grade 2 is mild degeneration, grade 3 is moderate, and grade 4 is severe degeneration. In the present study, the Pauli score was used as continuous measure; no conversion to grades was performed

1. Surface integrity	
Femoral surface	Score
• Smooth	0
• Slight fibrillation	1
• Moderate fibrillation	2
• Severe fibrillation	3
Tibial surface	
• Smooth	0
• Slight fibrillation	1
• Moderate fibrillation	2
• Severe fibrillation	3
Inner rim	
• Smooth	0
• Slight fibrillation	1
• Moderate fibrillation	2
• Severe fibrillation	3
2. Cellularity	
• Normal	0
• Hypercellularity	1
• Diffuse hypocellularity	2
• Acellular	3
3. Collagen organization/alignment and fiber organization	
• Collagen fibers organized	0
• Collagen fibers organized and foci of mucinous degeneration	1
• Collagen fibers unorganized and foci of mucinous degeneration	2
• Collagen fibers unorganized and fibrocartilaginous separation	3
4. Matrix staining (safranin O-fast green)	
• None	0
• Slight	1
• Moderate	2
• Strong	3

### Quantitative MR image analysis

**Fig. 2:**
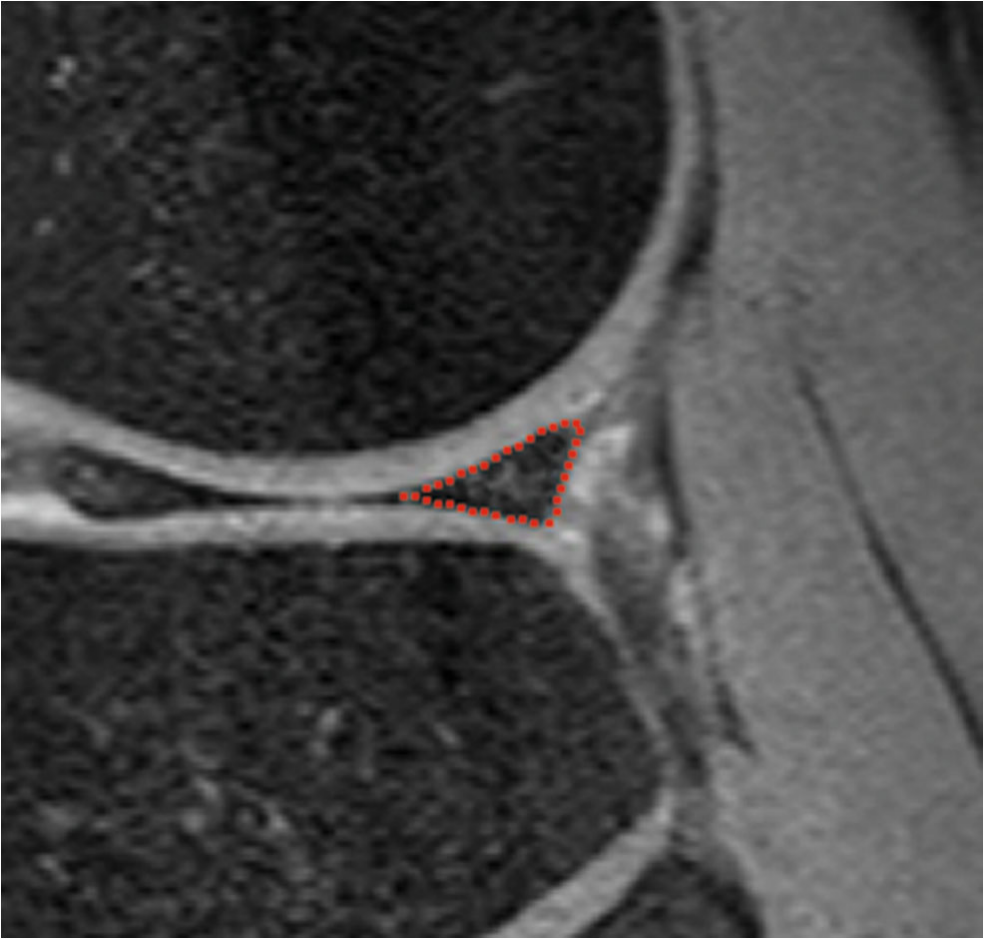
Representative example of non-contrast sagittal T_2_ image with manually drawn ROI of the posterior horn of the lateral meniscus in a 67-year-old female with knee osteoarthritis

On T_2_ mapping images, meniscal regions of interest (ROIs) were manually segmented by a researcher with a medical degree and 4 years of experience in musculoskeletal research (Fig. 2), who was blinded to patient information and histology outcomes. Meniscal segmentation was performed using an image collected with the echo time (TE) showing optimal contrast between menisci and surrounding tissues (TE 7.3 ms).

Great care was taken to match MR imaging ROIs and histological ROIs. As described earlier, histological tissue processing was performed using predefined anatomical regions: the most central part of the anterior horn and the most central part of the posterior horn. As histological samples were cut in a fixed and standardized way, MR imaging ROIs were matched to histological ROIs. To do so, we identified the most central slice through the medial and lateral meniscus (defined as the sagittal slice depicting the maximum width of the anterior horn and posterior horn as individual triangles) along with the neighboring slices medially and laterally. Four ROIs were defined per patient: the anterior and posterior horn of the medial and lateral meniscus. All ROIs consisted of three consecutive slices: the most central slice along with the adjacent slice medially and laterally. MR imaging scout views, using T_2_-weighted images in the coronal and axial plane, were used to verify that the ROIs were correctly defined (i.e., that they matched histological ROIs).

For MR image postprocessing, in-house developed registration and fitting algorithms in MATLAB (R2011a; The MathWorks) were used [17]. Automated rigid registration in 3D was used for motion compensation [17]. Similar to previous studies [18, 19], we excluded all images with TE above 30 ms because of the very low signal-to-noise-ratio in meniscal tissue (Table 1). To reduce effects of possible outliers within ROIs, T_2_ relaxation times were weighted by the reciprocal of the uncertainty of the estimated T_2_ relaxation time in each voxel. This uncertainty was measured with the square root of the Cramer-Rao lower bound, which gives a lower bound for the standard deviation of the estimated T_2_ relaxation time [17]. The weighted T_2_ mapping relaxation times for each ROI were averaged over the three consecutive MR imaging slices, further referred to as mean T_2_ relaxation time [17].

### Statistical analysis

Descriptive statistics for all available variables, including demographics, T_2_ relaxation times per meniscal ROI, and histological scores, are reported. Normality was tested using the Shapiro-Wilk tests. Normally distributed data were presented as mean with standard deviation; non-normally distributed data were presented as median with interquartile range (IQR).

Interobserver reliability of histological scoring was tested using two-way intraclass correlation coefficients (ICCs) of absolute agreement, taking single measurements.

We performed a linear mixed-effects model to assess the correlation between T_2_ relaxation times and histological scores, where T_2_ relaxation times were considered as dependent variable and histological score as independent variable. We employed the generalized least squares function in the “nlme” library in the statistical software “R” [20] allowing to calculate the correlation in repeated measures data (i.e., in datasets that include multiple measures per patient). Age, BMI, and sex were tested as potential covariates since they might impact T_2_ values. A backward variable selection and the likelihood ratio test were used for this purpose. Subgroup analyses were performed using a linear mixed-effects model, regarding regional differences.

Statistical analyses were performed using R version 3.4.2 (2017) [20].

## Results

### Patient characteristics

In total, 13 menisci were collected from 7 patients with knee OA: six medial and seven lateral menisci. There was a slight overall female predominance of 57%; the median age of patients was 67 years (range 59–74). None of the menisci showed a macroscopic tear. Patient characteristics are shown in Table 3.

**Table 3:** Characteristics of the study population, both of the total study population and stratified per sex

Characteristics of the study population	
No. of patients	7
No. of menisci	13
Age (years)*	67 (59–74)
Female patients	
No. of patients	4
Median age (years)	66
Age range (years)	59–67
Male patients	
No. of patients	3
Median age (years)	73
Age range (years)	66–74
Body mass index^†^ (kg/m^2^)	28 ± 4
Time interval between MR imaging and harvesting^†^ (days)	1 ± 0
Radiographic OA grade	KL grade 3: *n* = 3
	KL grade 4: *n* = 4
Most affected side of radiographic knee DA	Medial compartment: *n* = 6
	Lateral compartment: *n* = 1
Patients with meniscal tear	0

*OA* osteoarthritis, *KL* Kellgren and Lawrence

*Data are median values (range)

^†^Data are mean values ± standard deviation

### Radiographic knee OA

Patients had either moderate radiographic knee OA (KLG 3, *n* = 3) or severe radiographic knee OA (KLG 4, *n* = 4).

### T_2_ relaxation time in meniscal tissue

**Fig. 3:**
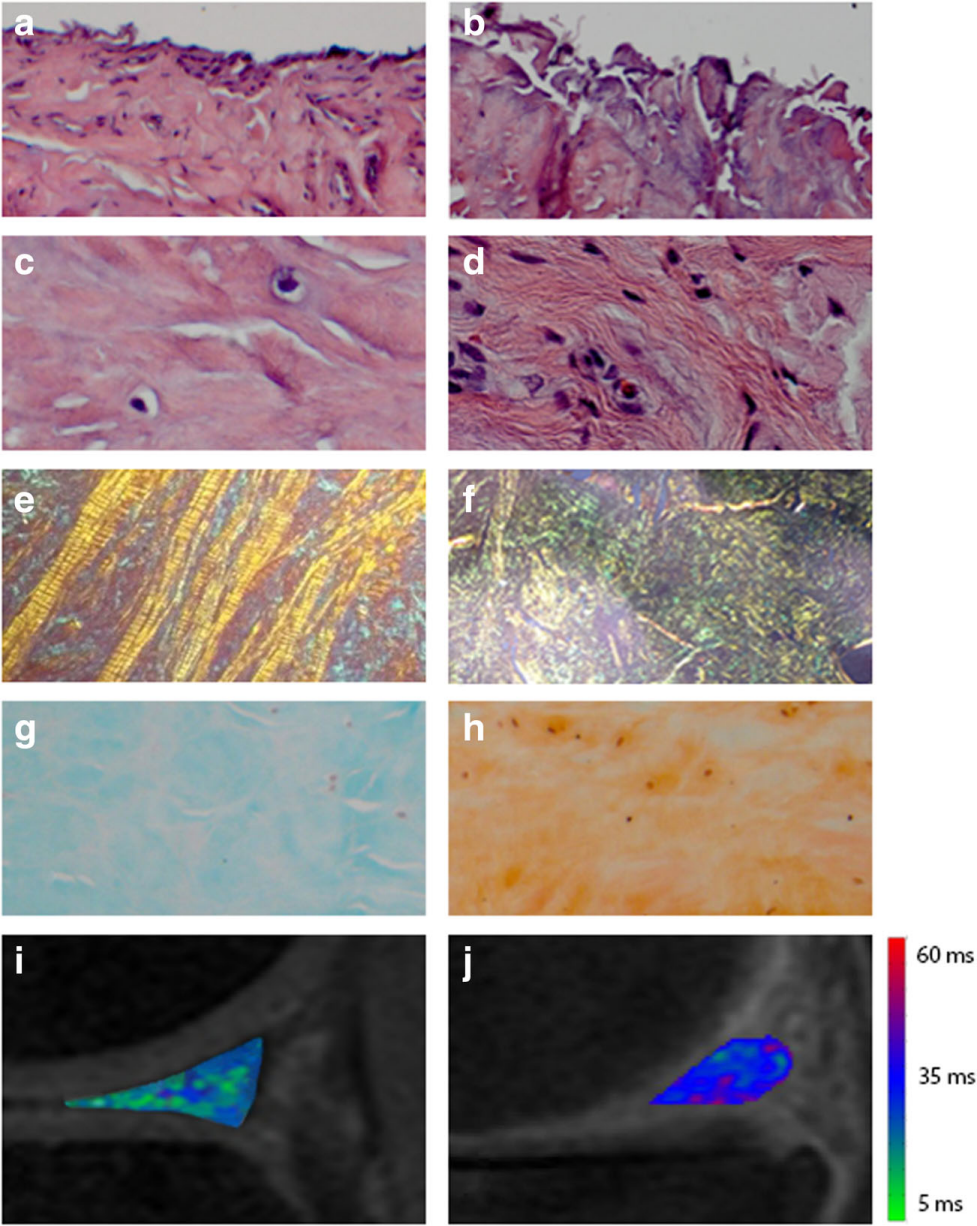
Representative images of histological findings in meniscal tissue and corresponding T_2_ mapping images. **a**, **c**, **e**, **g** Posterior horn of lateral meniscus of a 67-year-old female with knee OA (Kellgren and Lawrence grade 3), with a mean T_2_ relaxation time of 18.6 ms and a histological score of 5. **b**, **d**, **f**, **h** Posterior horn of medial meniscus of a 66-year-old female with knee OA (Kellgren and Lawrence grade 4) with a mean T_2_ relaxation time of 26.9 ms and a histological score of 13. **a**, **b** Surface integrity (HE staining, × 10 zoom). **c**, **d** Cellularity (HE staining, × 40 zoom). **e**, **f** Collagen organization (Picrosirius red staining, × 5 zoom). **g**, **h** Collagen matrix staining intensity, a decreased intensity of green staining indicates disruption in collagen matrix (Saf O-green staining, × 10 zoom). **i**, **j** Corresponding non-contrast sagittal T_2_ mapping images with color map of the meniscus. The color bar on the right shows the range of T_2_ relaxation times

The mean meniscal T_2_ relaxation time was 22.4 ± 2.7 ms (range 18.5–27). In addition to overall mean T_2_ relaxation times (i.e., the mean of measurements from all ROIs), mean T_2_ relaxation times were calculated for the meniscal ROIs (medial anterior and posterior, lateral anterior and posterior) separately, reported in Table 4. Only ROIs of which the corresponding histological meniscal region was available were included in analyses. Highest T_2_ relaxation times were found in the medial anterior horn of the meniscus. Statistical significantly higher T_2_ relaxation times were found in the medial menisci than in the lateral menisci (*p* = 0.005). No statistically significant differences between the anterior and posterior meniscal horns in T_2_ relaxation time were found (*p* = 0.14). Representative T_2_ mapping findings are displayed in Fig. 3i, j.

**Table 4:** Meniscal T_2_ measurements and histological scores per ROI

	T_2_ (ms)*	Histological score^†^
Medial meniscus, anterior horn	25.4 ± 1.5	12, 11–12
Medial meniscus, posterior horn	23.2 ± 2.6	10, 8.5–11.5
Lateral meniscus, anterior horn	20.8 ± 1.4	7, 6–8
Lateral meniscus, posterior horn	19.9 ± 1.2	8, 5–8

*ms* milliseconds

*Data are mean values ± standard deviations

^†^Data are median values, interquartile range

### Histological findings in meniscal tissue

In two patients, all four meniscal regions (medial anterior, medial posterior, lateral anterior, and lateral posterior) could be harvested. In the remaining five patients, as a result of partial maceration of the menisci due to end-stage knee osteoarthritis or severe damage during surgery, not all four regions could be harvested (only three regions possible in four patients and a single region in one patient). In total, 21 out of 28 meniscal regions were used for histological analysis (medial anterior: *n* = 6, medial posterior: *n* = 5, lateral anterior: *n* = 5, lateral posterior: *n* = 5).

The interobserver reliability of histological scoring between the two observers was excellent (ICC 0.95, CI 95% 0.79–0.99). We found an overall median histological score of 10, IQR 7–11 (range 4–13). Mean histological scores per meniscal ROI are shown in Table 4. As for T_2_ relaxation times, the highest histological scores were found in the medial anterior horn of the meniscus and histological scores were found to be higher in the medial menisci than in the lateral menisci (*p* = 0.007). Also, no statistically significant differences between the anterior and posterior meniscal horns in histological score were found (*p* = 0.20). Representative histological findings are displayed in Fig. 3a–h.

### Correlation between T_2_ mapping and histological scores

**Fig. 4:**
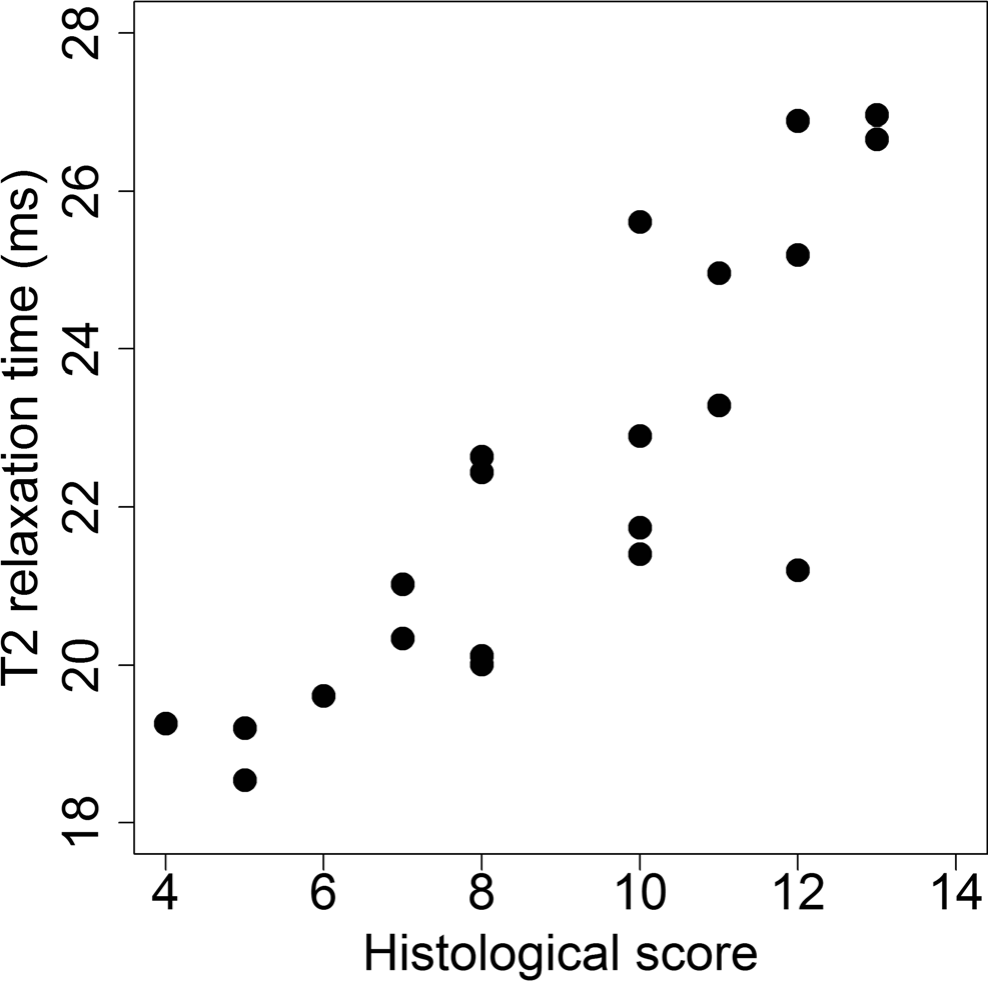
Scatterplot of histological scores versus T_2_ relaxation times in all patients and all measurements

In the linear mixed-effects model, the variables age, sex, and BMI were not statistically significant and were excluded from the model. To incorporate the potential effect of repeated measures (i.e., multiple measures per patient), the model has been statistically adjusted. A strong correlation between T_2_ mapping and histology (correlation coefficient 0.85, CI 95% 0.68–0.93) was found (Fig. 4).

## Discussion

In this study, we assessed the correlation between in vivo meniscal T_2_ mapping and histology in patients with radiographic knee OA. We demonstrated that meniscal T_2_ relaxation times in patients with knee OA show a strong correlation with the degree of histological degeneration. These findings indicate the potential of T_2_ relaxation times, obtained with in vivo T_2_ mapping, as non-invasive imaging biomarker for meniscal degeneration.

The results of our study are in line with those of previous research on meniscal T_2_ mapping where no histological analysis was performed. These studies showed that T_2_ mapping can differentiate between healthy patients and those with knee OA. Zarins et al found that meniscal T_2_ mapping discriminated between healthy and severe OA, but not between healthy and mild OA, and only in the posterior meniscal horns [19]. Rauscher and colleagues validated meniscal T_2_ mapping and T_1_rho against radiographical and clinical OA in subjects without OA, mild OA, and severe OA. They observed significant differences in T_2_ and T_1_rho values between subject groups and concluded that T_2_ mapping was more useful than T_1_rho for differentiating subject groups [13]. In addition to OA patients, T_2_ mapping has been investigated in patients with acute knee injury. Significantly higher T_2_ relaxation times were reported in patients with an anterior cruciate ligament rupture, compared with those in healthy controls [12]. To our knowledge, this is the first study to investigate the validity of in vivo meniscal T_2_ mapping in osteoarthritic patients, using histology as the reference test. Recently, Nebelung et al performed a comprehensive validation study of multiple quantitative MR imaging techniques, including T_2_ mapping, T_1_rho, and ultrashort echo time-enhanced T_2_* (UTE T_2_*) [7]. Histological analysis of meniscal samples from total knee replacement surgeries was used as the reference standard. In their study, strongest correlations between qMRI values and histology were found for T_2_ mapping and UTE T_2_*. In contrast to the present study, their T_2_ mapping measurements were performed ex vivo. Whether T_2_ measurements, obtained ex vivo, reflect the actual in vivo situation could be questioned. Several factors in ex vivo experiments may affect T_2_ relaxation times. First, storage of meniscal samples in medium and changes in tissue hydration may have potentially affected T_2_ measurements [7, 14]. Second, in ex vivo experiments, samples are typically scanned at room temperature and not at body temperature, potentially influencing T_2_ relaxation times. Last, ex vivo quantitative MR imaging experiments usually have different acquisition parameters, such as the number and duration of echo times, field of view, and acquisition matrix, compared with in vivo experiments [7, 21]. In addition to the differences between ex vivo and in vivo measurements, Nebelung and colleagues used a simplified, non-validated version of the Pauli score (the Williams score) to assess histological degeneration. These factors may have caused the lower correlation coefficient (*r* 0.65) between T_2_ mapping and histology in their study compared to ours.

T_2_ relaxation times have been increasingly used to assess meniscal tissue composition [7, 13, 14, 19], yet concerns have been raised that meniscal T_2_ mapping can be challenging due to the short T_2_ components and the heterogeneity of meniscal tissue [22–24]. In general, multi-echo T_2_ mapping sequences for knee OA have echo time (TE) values ranging from 10 to 100 ms [25], and mean T_2_ relaxation times of healthy menisci have been reported to be 11 ± 4 ms [13]. In previous studies, it has therefore been suggested that quantitative MR imaging techniques that obtain extremely short echo times, such as UTE T_2_*, may be more suitable to quantify menisci than standard spin-echo-based T_2_ mapping [23, 26, 27]. In the earlier mentioned study by Nebelung and colleagues, correlations between UTE T_2_* values and histology were comparable with correlations between T_2_ values and histology. However, they state that their choice of TE may not have been optimal for T_2_ mapping of the meniscus: acquired TE values ranged from 10 to 160 ms (TE 20–60 ms were used for analysis). Also, a 2D sequence was used for T_2_ mapping while a 3D sequence was used for UTE T_2_*, and single-slice quantitative analysis was performed, which may have influenced MRI outcomes and correlations [28]. In the present study, we took great care optimizing T_2_ mapping sequence parameters. We used a 3D spin-echo-based T_2_ mapping sequence with TE values ranging from 3 to 68 ms (TE 3–27 ms were used for analysis) and performed multi-slice quantitative analyses. We found a promising correlation between T_2_ values and histology (*r* 0.84, CI 95% 0.64–0.93), suggesting that in vivo spin-echo-based T_2_ mapping can provide accurate T_2_ measurements in menisci.

The results of the present study suggest that T_2_ relaxation times, obtained with in vivo T_2_ mapping, can potentially be used as non-invasive biomarker to detect early changes in meniscal tissue that indicate degeneration. T_2_ mapping allows a relatively wide range of TEs, with TE values short enough to assess menisci but long enough to assess articular cartilage [29–31]. Therefore, it may be a promising technique to detect early changes in various tissues involved in OA. The detection of early tissue changes, indicating degeneration, would allow a better understanding of the etiology and development of knee OA. Furthermore, it would allow the identification of patients at early OA stages, before irreversible damage occurs. Also, it would improve the monitoring of disease progression and treatment response. The long-term goal would be to allow the detection and monitoring of early tissue changes that indicate an increased risk for knee OA, potentially enabling early treatment strategies for knee OA.

This study has several limitations that should be considered. First, we had a limited sample size. Although a strong correlation was found between T_2_ values and histology, the small sample size may have impeded our statistical power. It should be noted, however, that meniscal degeneration was quite variable within the study population; the included menisci showed a relatively wide range of T_2_ values and histological score. Another limitation of the present study is that the meniscal body was not evaluated. The results of the present study may therefore only be generalizable for the meniscal horns. Also, we did not differentiate between meniscal zones (e.g., radially inner versus radially outer zone). The Pauli scoring system, which we used for histological grading of meniscal degeneration, does not distinguish meniscal zones. As the entire cross section needs to be assessed, a separate score for different meniscal zones is not possible. Finally, we could not include all meniscal regions of all menisci, as a result of complete maceration and/or severe damage during surgery. This issue should be considered for generalizing the results of this study. Future studies with greater sample size, and with further anatomical and zonal differentiation, should be conducted to reproduce our study results.

In conclusion, in vivo T_2_ mapping of the human meniscus provides accurate measurements of meniscal degeneration in patients with knee osteoarthritis. By quantifying subsurface meniscal changes, T_2_ mapping potentially provides a non-invasive imaging biomarker for meniscal degeneration.

## Electronic supplementary material

ESM 1 (DOCX 17 kb)

## Abbreviations

BMI: Body mass index
CI 95%: 95% confidence interval
Fat-Sat: Fat saturation
FSE: Fast spin echo
ICC: Intraclass correlation coefficient
IQR: Interquartile range
KLG: Kellgren and Lawrence grade
MR: Magnetic resonance
OA: Osteoarthritis
ROI: Region of interest
T: Tesla
TE: Echo time
