# Supplementary material for: T2 mapping of the meniscus is a biomarker for early osteoarthritis
Source: Eur Radiol. 2019 Mar 19;29(10):5664–72. doi: 10.1007/s00330-019-06091-1 (PMC6719322; doi:10.1007/s00330-019-06091-1)
Supplement: Supplementary file 1 — (DOCX 17 kb) [file 330_2019_6091_MOESM1_ESM.docx]

**Appendix 1: Assessment of radiographic knee osteoarthritis**

The degree of radiographic knee osteoarthritis was graded according to the Kellgren and Lawrence (KL) classification system [1], ranging from 0 (no OA) to 4 (end stage OA). The KL classification includes the assessment of joint space narrowing, osteophytes, subchondral sclerosis, and deformity of bone contour. Grading was performed by a musculoskeletal radiologist with 12 years of experience, using weight bearing anteroposterior radiographs. Radiographs and MR imaging scans were acquired on the same day.

**References:**

1 Kellgren JH, Lawrence JS (1957) Radiological assessment of osteo-arthrosis. Annals of the Rheumatic Diseases 16:494-502
